# Editorial: Artificial Intelligence in Bioinformatics and Drug Repurposing: Methods and Applications

**DOI:** 10.3389/fgene.2022.870795

**Published:** 2022-03-17

**Authors:** Pan Zheng, Shudong Wang, Xun Wang, Xiangxiang Zeng

**Affiliations:** ^1^ Department of Accounting and Information Systems, University of Canterbury, Christchurch, New Zealand; ^2^ College of Computer Science and Technology, China University of Petroleum, Qingdao, China; ^3^ Department of Computer Science, Hunan University, Changsha, China

**Keywords:** artificial intelligence, neural network, bioinformatics, drug repurposing, drug design

## Introduction

The development of Artificial Intelligence (AI) pushes the boundaries of new computing paradigms to become actual realities for many science and engineering challenges. The delicacy and agility of a computing instrument do not mean anything when we cannot use it to create value and solve problems. Machine learning, a trendy subfield of AI, focuses on extracting and identifying insightful and actionable information from big and complex data using different types of neural networks. Data-hungry by its nature, machine learning algorithms usually excel in the practical fields that generate and possess abundant data. The two application areas we are interested in are drug repositioning and bioinformatics, both of which are the very fields often producing a large volume of data. This thematic issue aims to cover the recent advancement in artificial intelligence methods and applications that are developed and introduced in the field of bioinformatics and drug repurposing and provide a comprehensive and up-to-date collection of research and experiment works in these areas.

## Bioinformatics

Bioinformatics is an interdisciplinary field that covers a broad spectrum of studies including fields of biology, computer science, information science, and statistics. DNA and gene data sequencing (Zou et al., 2016), coding (Yin et al., 2021), modification (Li et al., 2015; Shi et al., 2016), and structure analysis (Zhang et al., 2014; Hong et al., 2020) are predominant areas of bioinformatics. This issue includes six contributions to this topic.

DNA sequences react with each other due to complementary pairing, which reduces the number of DNA sequences that could be used for molecular hybridization. If the A-T base is located at one end of the DNA sequence and its complementary sequence, it results in a gap and leads to the decreasing of the accuracy of the calculation. Pairing Sequences Constraint (PSC) and Close-ending along with the Improved Chaos Whale (ICW) optimization algorithm are proposed by Li et al. in this Research Topic to effectively resolve the issues. The proposed method is compared with the contemporary methods of NACST/Seq, DEPT, MO-ABC, pMO-ABC, and HSWOA on parameters such as continuity, hairpin structure, H-measure, similarity, and melting temperature. The experiment shows that the method demonstrates superior results and the ability to avoid secondary structures.

The volume of the data is growing exponentially in the big data era. Data storage is becoming a pressing challenge. The unique design and molecular structure of deoxyribonucleicacid (DNA) provide us with a mechanism to encode and store humongous amounts of data very efficiently. Zheng et al. present an algorithm of coding sets for DNA storage. It is essentially a variant of Gradient-Based Optimizer (GBO) with two mutation strategies for better performance, which are the Cauchy and Levy mutation operators. The performance of the algorithm was evaluated using CEC-2017 test function and the Wilcoxon rank-sum test. It showed that the lower bounds of DNA coding sets constructed by the CLGBO algorithm increased by 4.3–13.5% compared with previous work.

Feng et al. looked at an interesting application of utilizing gene and DNA data for cancer subtype discovery and related gene identification. The study experimented on the data collected from the Broad Institute GDAC Firehose, which contains seven common cancer datasets. Each cancer dataset consists of multiple sources of cancer data including gene expression information, isoform expression information, DNA methylation expression information, and corresponding clinical information. The raw data was initially rescaled by min-max normalization and reduced by kernel PCA. Based on the multi-omics of cancer data, three similarity kernel matrices are constructed through the Gaussian kernel function and fused into a global similarity expression matrix. Eventually, the integrated similarity kernel matrix is fed to spectral clustering, hence the predictive clusters are identified. The performance of the approach demonstrates reasonable superiority in comparison with other similar methods.

Another study of gene data by Ding et al. focuses on identifying modules and characteristic genes of pancreatic cancer. A non-NMF network analysis method (NMFNA) is introduced. Initially, the methylation network (ME), copy number variation (CNV) network, and ME-CNV network are constructed by Pearson correlation coefficient. Incorporating graph-regularized constraints, the networks are further integrated and decomposed to identify modules. Both gene ontology (GO) and pathway enrichment analyses are performed, and characteristic genes are detected by the multi-measure score to understand the biological functions of PC core modules. Compared with similar methods in the literature, the NMFNA identified more PC-related GO terms, pathways, and characteristic genes in core modules during experiments.

The intrinsic nature of the DNA structure inspires the studies of other fields, e.g., computing. Xue et al. developed an image protection algorithm based on the chain of the dynamic length of DNA. The method is tested on images generated by three popular medical imaging modalities: CT, MRI, and X-Ray. In this method, the original image is encoded into a DNA matrix dynamically using a Fractional-Order Chen Hyper Chaotic (FOCHC) sequence, The DNA matrix is then scrambled by two other FOCHC sequences. DNA dynamical chain operations are carried out by four FOCHC sequences. To decode, the matrix is solved into a binary matrix by a FOCHC sequence, and the encrypted image is obtained after recombining the DNA chain. Eight chaotic sequences are used to complete the whole process. The eight sequences are generated by two FOCHC under different keys, which are produced using the SHA-256 algorithm and the hamming distance. The method is robust and able to be sustained under noise attack, occlusion attack, and all common cryptographic attacks.

Taking advantage of advanced computational methods to analyze DNA data conveys great value in the area of bioinformatics. Yang et al. propose a method that can identify DNA modification sites, N4-methylcytosine (4 mC) and N6-methyladenine (6 mA). The method uses multitask learning integrated with the bidirectional gated recurrent units (BGRU). The original DNA sequences are encoded into matrices by one-hot encoding as the input of the neural network. The encoded matrix is fed to a bidirectional GRU for the different levels of dependency relationships between subsequences. A max-pooling layer is used to find out features playing key roles in DNA methylation site identification in each GRU. The features learned from the max-pooling layer are sent to the task-specific output module to identify DNA modification sites. DNA datasets of four species are experimented with using the method. The performance is significantly better than the other methods using typical text classification.

## Drug Repurposing

Drug repurposing is an important topic in the research field of drug discovery and design. it is also known as drug repositioning, reprofiling, or re-tasking (Pushpakom et al., 2019), which is a technique or process of finding novel pharmaceutical applications of existing medicines that are not originally designed for. There are usually three types of studies to develop and examine the efficacy of a drug during the process of drug discovery and design, *in silicon*, *in vitro*, and *in vivo*. With artificial intelligence and machine learning in the role, the researches mainly focus on *in silicon* studies and some *in vitro* for proof of concept. With an increasing number of related drug databases, e.g., DrugBank, DrugMatrix, BindingDB, PubChem, ChEMBL, and KEGG to name a few, drug data analysis and study using artificial neural networks and other machine learning methods become the trend in computer-aided drug design. There are three major types of conventional studies which are Drug-Target Interaction (DTI), Drug-Drug Interaction (DDI), and Protein-Protein Interaction (PPI). Drug-Target Interaction (DTI) is the most popular type of study in this field. It investigates the binding relation, i.e., binding affinity, between the drug and target protein directly. The early studies consider DTI a binary classification problem and later various machine learning methods are used to approach DTI problems (Öztürk et al., 2018; Song et al., 2021). Drug-Drug Interaction (DDI) studies explore the effect variations of a drug when the drug is taken at the same time with another (Kumar Shukla et al., 2020). Drug interaction profiles can be established to measure drug similarities and associations. It is believed that drug molecules with homogenous structures are probable to react on similar proteins and manifest similar physiological effects. Protein-Protein Interaction (PPI) is another type of study that probes into the drug discovery problem. PPI exists in all the biological and cellular processes, e.g., cell-signaling and cell survival. In drug discovery, finding allosteric sites and hotspots has become a popular topic of PPI studies (Liu et al., 2018; Jin et al., 2021; Yu et al., 2022). Another six contributions of drug repurposing are included in this issue.

Alzheimer’s disease (AD) is common dementia that develops among the elderly. The mitochondrial fusion protein 2 (MFN2) is one of the closely relevant proteins which may cause AD. Wang et al. develop a three-tunnel deep neural network model trained on the Davis dataset and deployed it with the DrugBank database to investigate the drug-target binding affinities between drug molecules and MFN2. Fifteen drug molecules were recommended by the neural network model. Molecular docking experiments were carried out on 11 of those whose molecular weights are greater than 200. The result shows that all 11 molecules can dock with the protein successfully and five of them have a great binding effect. This work demonstrates a classical approach of DTI using neural network methods.

Liu et al. propose a novel DTI prediction method (GADTI) using graph convolutional network (GCN) and a random walk with restart (RWR). Data used in this study are first converted in the form of a graph network. DTI predictions are then transformed into link predictions of the network. The overall architecture comprises two main components, an encoder and a decoder. The encoder is formed by the GCN and the RWR, which produces embeddings for nodes of the graph. The decoder is a matrix factorization model using embedding vectors from the encoder to discover and predict DTIs. Experiments show that GADTI has an AUROC value of 0.9582 and AUPRC of 0.8611. Four popular DTI methods are used as benchmarking methods, all of which are inferior to GADTI. It is quite refreshing that the researchers endeavor to explore new neural network methods in the DTI of drug discovery and repurposing.

As graph neural networks (GNNs) gain popularity in solving various practical problems, Zhang et al. present a comprehensive survey of GNNs and their advances in bioinformatics. Three major variants of GNN, Graph Convolutional Networks, Graph Attention Networks, and Graph Autoencoder Networks are reviewed. Typical technical tasks of GNN, node classification, link prediction, and graph generation, are thoroughly discussed. The applications of GNN in literature are categorized in three bioinformatics application aspects, disease prediction, drug discovery, and biomedical imaging. Besides the merits of GNN, the survey addresses the challenges of GNN as well, e.g., data quality and method Interpretability. We noticed that there are several surveys of GNN published from 2018 to 2021, nonetheless, a dedicated GNN survey in the field of bioinformatics was lacking. The contributors of this work timely fill the gap. It has been cited 8 times in the first 6 months of its publication, thus we believe this work will attract more research attention in the future.

How to produce new reasonable molecules with desired pharmacological, physical, and chemical properties is one of the challenges of *de novo* molecular generation. Wang et al. develop a new variant of cycle generative adversarial network (CycleGAN) named LA-CycleGAN which is embedded with Long Short-Term Memory (LSTM) and Attention mechanism for molecule generation with better accuracy. With the new mechanisms added, the neural network is able to overcome long-term dependency problems in treating the commonly used SMILES input. The quantitative evaluation and experiments show that LA-CycleGAN achieves better Tanimoto similarity distribution between the generated molecules and the starting molecules in comparison with a similar variant, Mol-CycleGAN. This study focuses on a specific machine learning method and improves it so the method can better work with molecular generation problems.

Disease module identification is an important step to potential drug targets formation. Liu et al. put forward an effective disease module identification method, IDMCSS. Modifying an existing PPI network, the method adds some potential interactions and removes incorrect interactions based on the connective and semantic similarities between the given disease proteins and their neighboring proteins. The method aims to eliminate the interference of incorrect and missing links contained in the original PPI network for better disease module detection. The method is experimented on an asthma PPI network and compared with four state-of-the-art disease module identification approaches. The disease module identified by IDMCSS includes more proteins that are enriched in asthma-related GO terms, pathways, and differential expression genes than those achieved by the other four approaches.

Drug repurposing against COVID-19 attracts wide research attention in the community. Harigua-Souiai et al. propose a pipeline for Ligand-Based Drug Discovery (LBDD) against SARS-CoV-2. A dataset of 2,610 molecules having anticoronavirus effects is collected and curated. The chemical structures of these molecules were encoded through multiple systems to be readily useful as input of a set of AI methods. Seven machine learning (ML) algorithms and four deep learning (DL) algorithms were used to classify the molecules in active and inactive classes. The seven ML algorithms are Logistic Regression (LR), Support Vector Machine (SVM), Random Forests (RF), Multitask Classifier (MTC), IRV-MTC, Robust MTC, and Gradient Boosting (XGBoost). Four DL methods are the Graph Convolutional Model, the DAG model, the Graph Attention Networks model (GAT), and the GCN model. The Random Forests (RF), Graph Convolutional Network (GCN), and Directed Acyclic Graph (DAG) models achieved the best performances. A further validation experiment revealed a superior potential of DL algorithms to achieve drug repurposing against SARS-CoV-2 based, i.e., GCN and DAG. This study exhaustively used almost all suitable Al algorithms and methods for the problem and suggested that DL methods are superior to others.

## Conclusion

After the special thematic issue information was announced as a “research topic” on the webpage of Frontiers in Genetics journal, it received overwhelming attention and interest from researchers in the field of bioinformatics and drug discovery. After careful and rigid reviewing and selection process, 12 papers were eventually selected. It is a very successful collection of contributions. At the time of writing this editorial, i.e., 12 months after the first accepted paper and 2 months after the last accepted paper was published, the research topic has received 25 citations, nearly 5000 downloads, and 27,000 views.
